# Morphological and molecular data reveal a new spider species of the little-known *Pholcus nagasakiensis* species group (Araneae, Pholcidae) from the Ryukyu Archipelago

**DOI:** 10.3897/BDJ.14.e193642

**Published:** 2026-04-29

**Authors:** Meichen Yan, Minghui Li, Francesco Ballarin, Zhiyuan Yao

**Affiliations:** 1 College of Life Science, Shenyang Normal University, Shenyang 110034, Liaoning, China College of Life Science, Shenyang Normal University Shenyang 110034, Liaoning China; 2 Department of Zoology, Museo di Storia Naturale of Verona, Lungadige Porta Vittoria, 9, I-37129, Verona, Italy Department of Zoology, Museo di Storia Naturale of Verona Lungadige Porta Vittoria, 9, I-37129, Verona Italy; 3 Systematic Zoology Laboratory, Department of Biological Sciences, Tokyo Metropolitan University, 1-1 Minami-Osawa, Hachioji-shi, 192-0397, Tokyo, Japan Systematic Zoology Laboratory, Department of Biological Sciences, Tokyo Metropolitan University 1-1 Minami-Osawa, Hachioji-shi, 192-0397, Tokyo Japan

**Keywords:** biodiversity, DNA barcode, East Asia, invertebrate, molecular species delimitation, Ryukyu Archipelago, taxonomy

## Abstract

**Background:**

The *Pholcus nagasakiensis* species group is a relatively small group, comprising only five species. Of these, four species inhabit Kyushu Island in mainland Japan, the Ryukyu Archipelago and the Diaoyu Islands of China, while one additional species has been documented from Wuyishan Mountain in mainland China.

**New information:**

Three morphologically similar species from the Ryukyu Archipelago, belonging to the *Pholcus nagasakiensis* species group were identified, based on an integrative approach combining morphology and molecular species-delimitation methods. These comprise one new species, namely *Pholcus yajiyagama* Yan, Ballarin & Yao, **sp. nov**., herein described, based on both sexes and two previously described species, *P. nagasakiensis* Strand, 1918 and *P. otomi* Huber, 2011. DNA barcodes were obtained for all three species to estimate p-distances and K2P distances and confirm their identifications. New diagnoses and detailed photomicroscopy images of the two previously described species, as well as a distribution map of all the species discussed, are also provided.

## Introduction

*Pholcus* Walckenaer, 1805 is the most diverse genus within the family Pholcidae C.L. Koch, 1850. It is primarily distributed across the Afrotropical, Palaearctic, Indo-Malayan and Australasian Regions (Huber 2011, Yao and Li 2012, Yao and Li 2025, Zhang et al. 2025, WSC 2026). This genus comprises 21 species groups and 434 valid species (Huber 2011, Huber et al. 2018, WSC 2026). In recent years, a series of surveys on *Pholcus* have been conducted in Asia. However, these efforts have mainly focused on the *P. phungiformes*, *P. bidentatus*, *P. crypticolens*, *P. yichengicus* and *P. taishan* groups from China, as well as the *P. phungiformes* group from Korea (Lee et al. 2021, Yao et al. 2021, Lu et al. 2022, Jang et al. 2023, Zhao et al. 2023, Lee et al. 2024, Yang et al. 2024a, Yang et al. 2024b, Jang et al. 2025, Lee et al. 2025, Li et al. 2025a, Li et al. 2025b).

**Figure 1.**
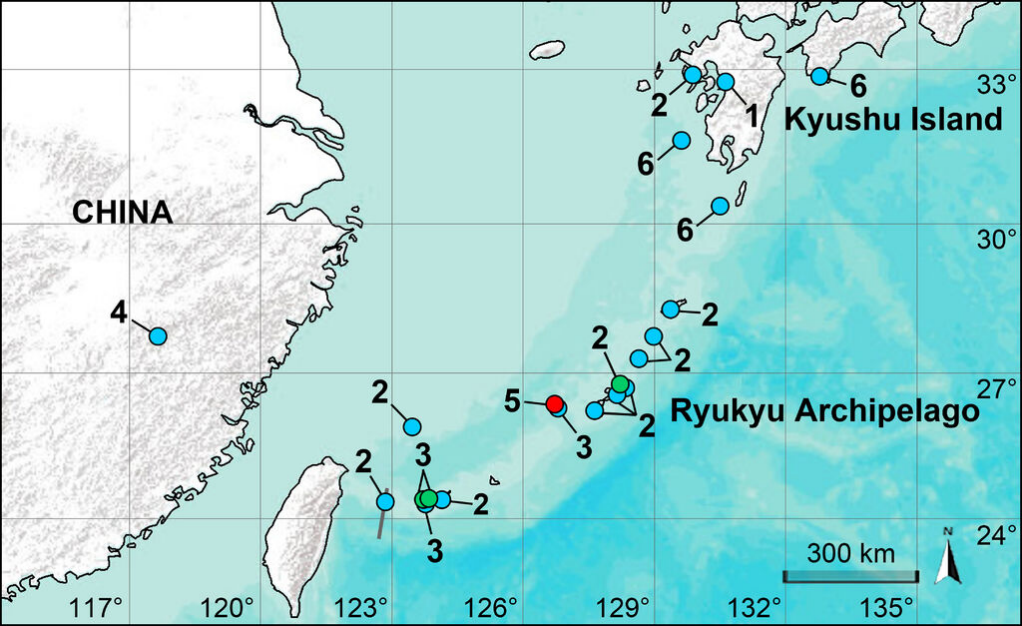
Distribution records of species of the *Pholcus nagasakiensis* species group. **1** *P. higoensis*; **2** *P. nagasakiensis*; **3** *P. otomi*; **4** *P. wuyiensis*; **5** *P. yajiyagama* sp. nov.; **6** *P. yoshikurai*. Blue, green and red circles represent previously recorded species, known species collected in this study and new species, respectively.

The *Pholcus nagasakiensis* species group is a relatively small group, distributed exclusively in East Asia, consisting of only five species (Huber 2011, WSC 2026). Amongst them, four species are found in Kyushu Island in mainland Japan, the Ryukyu Archipelago and the Diaoyu Islands of China, while one additional species is recorded from the Wuyishan Mountain in mainland China. Based on morphological and molecular evidence, this paper identifies three morphologically similar species from the Ryukyus, including one undescribed species, all of which belong to the *P. nagasakiensis* group (Fig. 1).

## Materials and methods

*Pholcus* individuals were hand-collected during surveys organised by the Systematic Zoology Laboratory of the Tokyo Metropolitan University, Japan (TMU), in the Ryukyu Archipelago and preserved in pure ethanol for morphological and molecular studies. Specimens were examined and measured with a Leica M205 C stereomicroscope and a Nikon SMZ1270 in the laboratories of the Shenyang Normal University, China (SYNU) and the Tokyo Metropolitan University, Japan, respectively. Left male palps were photographed. Epigynes were photographed before dissection. Vulvae were illustrated after treating them in a 10% warm solution of potassium hydroxide (KOH) to dissolve soft tissues. Images were captured with a Canon EOS 750D wide zoom digital camera (24.2 megapixels) mounted on the stereomicroscopes mentioned above and assembled using Helicon Focus v. 3.10.3 image stacking software (Khmelik et al. 2005). All measurements are given in millimetres (mm). Leg measurements are shown as: total length (femur, patella, tibia, metatarsus, tarsus). Leg segments were measured on their dorsal side. The distribution map was generated with ArcGIS v. 10.2 (ESRI Inc.). The specimens studied are deposited in the College of Life Science, Shenyang Normal University in Liaoning, China and the National Museum of Nature and Science (NSMT), in Tsukuba, Japan.

Terminology and taxonomic descriptions follow Huber (2011), Yao et al. (2015) and Yao et al. (2021). The following abbreviations are used: **a** = appendix, **aa** = anterior arch, **ALE** = anterior lateral eye, **AME** = anterior median eye, **b** = bulb, **da** = distal apophysis, **dp** = distal process, **ds** = dorsal spine, **dsp** = dorso-subdistal process, **e** = embolus, **kn** = knob, **L/d** = length/diameter ratio, **mb** = median branch, **me** = membranous edge, **pa** = proximo-lateral apophysis, **PME** = posterior median eye, **pp** = pore plate, **pr** = procursus, **pra** = proximal apophysis, **psa** = prolatero-subdistal apophysis, **u** = uncus, **va** = ventral apophysis, **vp** = ventral protrusion, **vsa** = ventro-subdistal apophysis.

The mitochondrial gene fragment encoding COI were obtained for seven samples (Table 1), using the following primers: forward: COIJerry 2 (5’-CAGCATTTGTTTTGATTTTTTGG-3’) and reverse: C1-N-2776 (5’-GGATAATCAGAATATCGTCGAGG-3’) (Simon et al. 1994). Two species *Pholcus paralinzhou* Zhang & Zhu, 2009 and *P. taishan* Song & Zhu, 1999 were selected as outgroups. DNA sequences were checked and edited with BioEdit 7.2.2 (Hall 1999). P-distances and K2P distances from COI were computed using MEGA 5 (Tamura et al. 2011). Phylogenetic trees were constructed using the Maximum Likelihood (ML) method for molecular species delimitation. ML analyses were conducted using RAxML 8.2.9 under a GTRCAT model for all partitions, with 500 rapid bootstrap replicates followed by a thorough Maximum Likelihood tree search (Stamatakis 2014). The sequences are deposited in GenBank. For additional information on extraction, amplification and sequencing procedures, see Yao et al. (2016) and Yao and Li (2025).

Three methods for molecular species delimitation were used. The Automatic Barcode Gap Discovery (ABGD) analyses were conducted using both Jukes–Cantor and Kimura 2-P distance matrices with options: Pmin = 0.001, Pmax = 0.1, Steps = 10, X = 1.0, Nb bins = 20 (Puillandre et al. 2012). The Bayesian implementation of the Poisson Tree Processes (bPTP) analysis was run for 100,000 generations, with a thinning of 100 and burn-in of 0.25 (Zhang et al. 2013). The Generalised Mixed Yule Coalescent (GMYC) analysis was performed under the single threshold model using the R 4.5.2 package SPLITS (R Development Core Team 2025). The phylogenetic tree was converted to an ultrametric format for GMYC analysis using BEAST 1.8.2 (Drummond et al. 2012).

## Data resources

**Figure 2.**
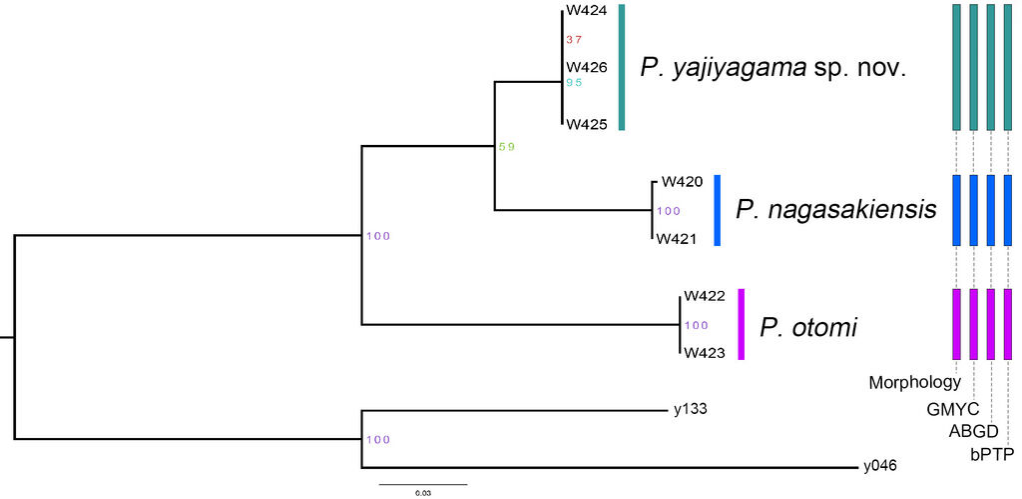
Results of the species delimitation analysis conducted using morphology, GMYC, ABGD and bPTP; different colours of the bars represent the different species. The phylogenetic tree was inferred from ML analysis, bootstrap values are provided at the nodes, branch lengths are scaled to the number of substitutions per site.

We obtained an alignment of 550 bp of COI gene. Fig. 2 displays the phylogenetic tree constructed from the COI data. The tree clearly divided the samples into three deeply divergent clades. We classified the three major clades as three candidate species because the ABGD, GMYC and bPTP analyses unambiguously support their status as separate species and the results are fairly consistent with morphological evidence. Furthermore, the smallest p-distance and K2P distance amongst these species is calculated as 0.059 and 0.062, respectively, between *P. nagasakiensis* and *P. yajiyagama* sp. nov. (Table 2). Amongst the three species, one is revealed to be new to science and its detailed description, based on both sexes, is provided herein. Additionally, new diagnoses and photomicroscopy images for the two known species are also presented. This species group can be recognised by Huber (2011).

## Taxon treatments

### Pholcus yajiyagama

Yan, Ballarin & Yao sp. nov.

212DF625-77AD-5D71-A25A-FD52E9D67275

C34B6501-7C17-4CC9-9239-8F74187EDCCA

#### Materials

- **Type status:** Holotype. **Occurrence:** recordedBy: Francesco Ballarin; individualCount: 1; sex: male; lifeStage: adult; occurrenceID: 5EC2E8F4-66D6-52C3-ABE9-E62B4BBD6C83; **Taxon:** order: Araneae; family: Pholcidae; genus: *Pholcus*; **Location:** island: Kume-jima Island; stateProvince: Okinawa Prefecture; county: Shimajiri District; municipality: Nakachi; verbatimLocality: near entrance of Yajiya-gama Cave; verbatimElevation: 42 m; verbatimLatitude: 26.36937°N; verbatimLongitude: 126.73337°E; **Event:** samplingProtocol: by hand; year: 2022; month: 5; day: 17; **Record Level:** institutionCode: SYNU-Ar00524
- **Type status:** Paratype. **Occurrence:** recordedBy: Koki Kamada; individualCount: 3; sex: 2 males, 1 female; lifeStage: 3 adults; occurrenceID: 699AA5CE-A22F-55AC-9EC2-6B98AB9FC296; **Taxon:** order: Araneae; family: Pholcidae; genus: *Pholcus*; **Location:** island: Kume-jima Island; stateProvince: Okinawa Prefecture; county: Shimajiri District; municipality: Nakachi; verbatimLocality: near entrance of Yajiya-gama Cave; verbatimElevation: 42 m; verbatimLatitude: 26.36937°N; verbatimLongitude: 126.73337°E; **Event:** samplingProtocol: by hand; year: 2025; month: 7; day: 26; **Record Level:** institutionCode: NSMT-Ar26764–26766

#### Description

**Male** (holotype): Measurements: Total length 7.31 (7.44 with clypeus), carapace 1.80 long, 2.13 wide, opisthosoma 5.51 long, 1.38 wide. Leg I: 52.09 (12.50, 0.86, 12.69, 23.07, 2.97), leg II: missing, leg III: 27.30 (7.76, 0.82, 6.70, 10.54, 1.48), leg IV: 35.43 (10.26, 0.83, 8.56, 13.85, 1.93); tibia I L/d: 58. Eye interdistances and diameters: PME–PME 0.28, PME 0.24, PME–ALE 0.06, AME–AME 0.10, AME 0.13. Sternum width/length: 1.36/1.28.

Colour: Carapace yellowish, with median brown marks; ocular area brownish, with median brown band; clypeus and sternum brown. Legs yellowish, but brown on patellae and whitish on distal parts of femora and tibiae, without darker rings on femora and tibiae. Opisthosoma yellowish, with dorsal and lateral brown spots.

**Figure 3.**
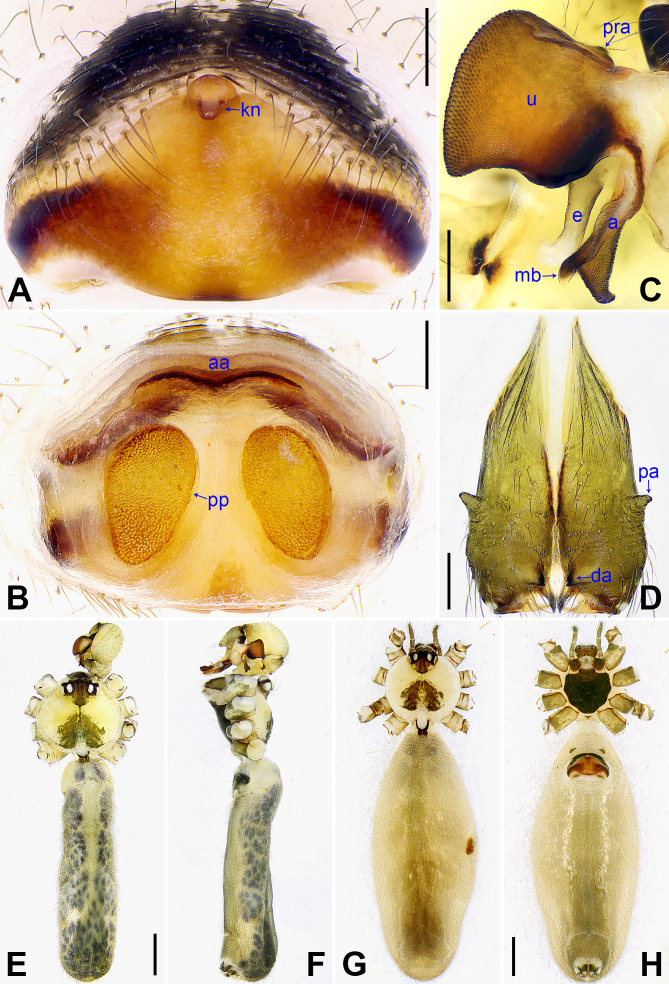
*Pholcus yajiyagama* sp. nov., holotype male (**C–F**) and paratype female (**A, B, G, H**). **A** Epigyne, ventral view; **B** Vulva, dorsal view; **C** Bulbal apophyses, prolateral view; **D** Chelicerae, frontal view; **E, G** Habitus, dorsal view; **F** Habitus, lateral view; **H** Habitus, ventral view. Scale bars: 0.20 mm (**A–D**); 1.00 mm (**E–H**).

Body: As in Fig. 3E and F; ocular area elevated, each eye triad on top of laterally directed eye-stalk.

Chelicerae: As in Fig. 3D, with pair of proximo-lateral apophyses (pa) and pair of distal apophyses (da) with two teeth each.

**Figure 4.**
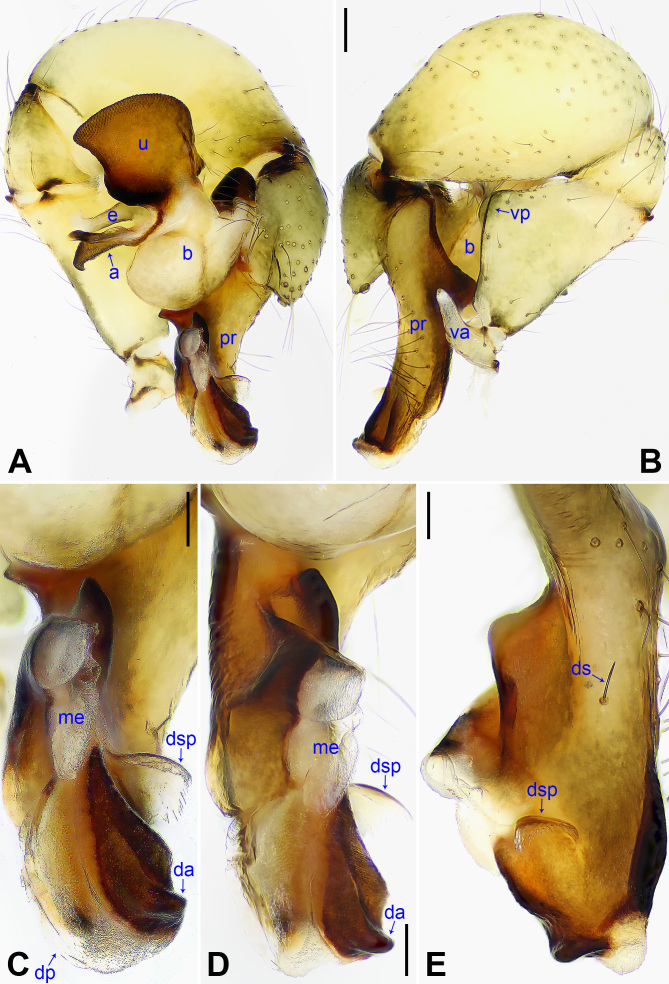
*Pholcus yajiyagama* sp. nov., holotype male. **A** Palp, prolateral view; **B** Palp, retrolateral view; **C** Distal part of procursus, prolateral view; **D** Distal part of procursus, prolateral-ventral view; **E** Distal part of procursus, dorsal view. Scale bars: 0.20 mm (**A, B**); 0.10 mm (**C–E**).

Palp: As in Fig. 4A and B; trochanter with long (2× longer than wide) ventral apophysis (va); femur with distinct ventral protrusion (vp); procursus (pr) simple proximally, but complex distally, with raised prolatero-subdistal edge bearing distal membranous process (dp), distal sclerotised apophysis (da), dorso-subdistal membranous process (dsp) and one slender dorsal spine (ds); uncus (u) distally blunt, with proximal apophysis (pra) and scales; appendix (a) hooked, with scales and median branch (mb); embolus (e) weakly sclerotised, with transparent distal projections.

Legs: Retrolateral trichobothrium on tibia I situated at 1.6% proximally; legs with short vertical setae on tibiae, metatarsi and tarsi; tarsus I with 22 distinct pseudosegments.

**Female** (paratype, NSMT-Ar26766): Similar to male, habitus as in Fig. 3G and H. Total length 8.57 (8.72 with clypeus), carapace 1.87 long, 1.96 wide, opisthosoma 6.70 long, 2.75 wide; tibia I: 11.24; tibia I L/d: 56. Eye interdistances and diameters: PME–PME 0.26, PME 0.20, PME–ALE 0.05, AME–AME 0.05, AME 0.10. Sternum width/length: 1.24/1.16. Opisthosoma without spots. Ocular area without eye-stalks. Epigyne (Fig. 3A) nearly triangular, with knob (kn). Vulva (Fig. 3B) with curved, sclerotised anterior arch (aa) and pair of nearly elliptical pore plates (pp).

#### Diagnosis

**Figure 5.**
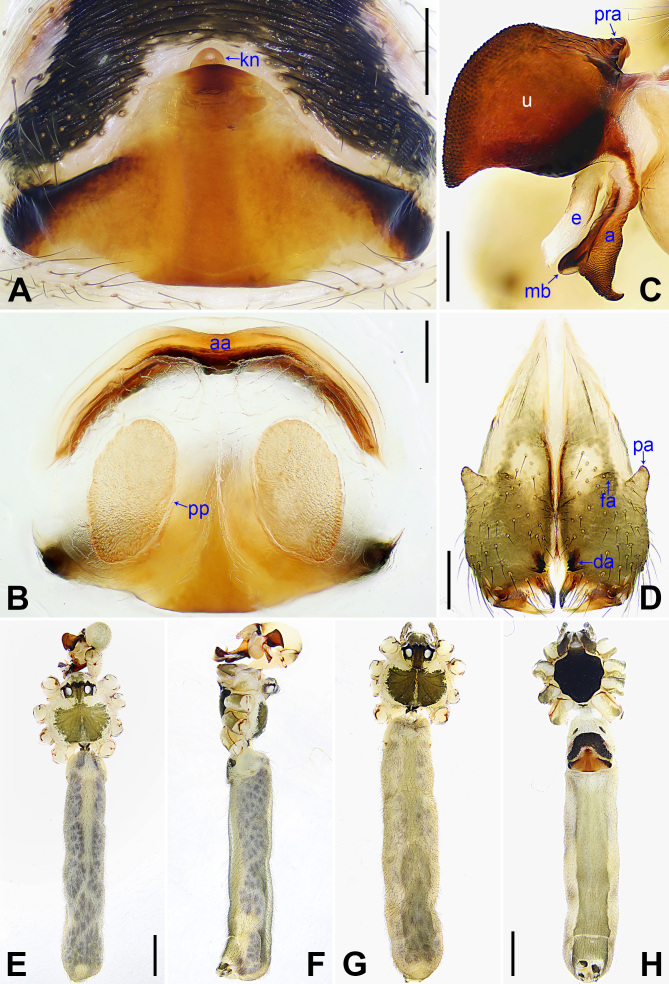
*Pholcus nagasakiensis* Strand, 1918, male (**C–F**) and female (**A, B, G, H**). **A** Epigyne, ventral view; **B** Vulva, dorsal view; **C** Bulbal apophyses, prolateral view; **D** Chelicerae, frontal view; **E, G** Habitus, dorsal view; **F** Habitus, lateral view; **H** Habitus, ventral view. Scale bars: 0.20 mm (**A–D**); 1.00 mm (**E–H**).

**Figure 6.**
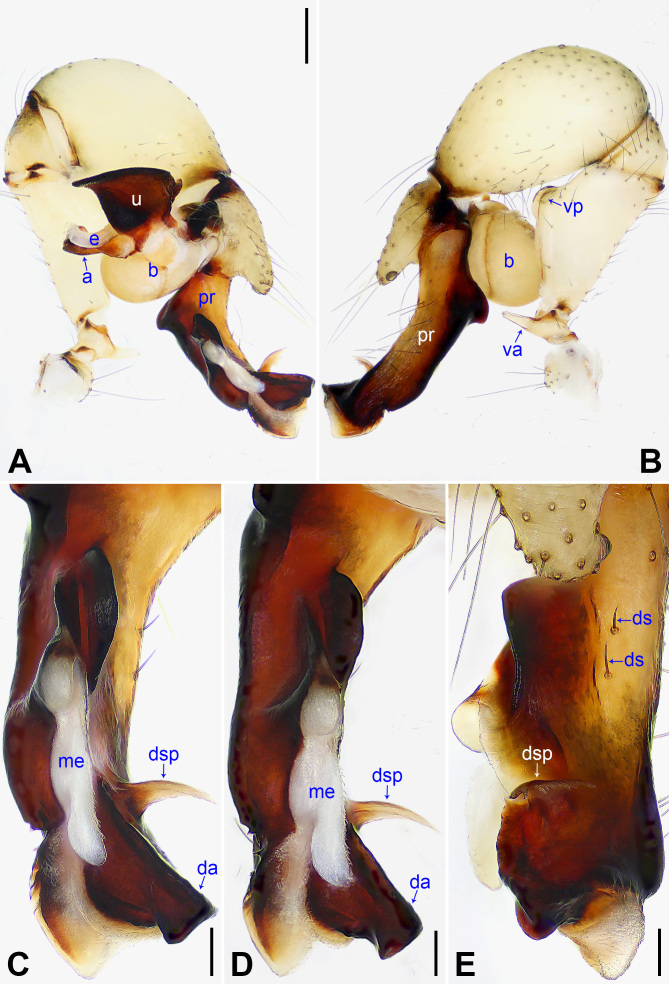
*Pholcus nagasakiensis* Strand, 1918, male. **A** Palp, prolateral view; **B** Palp, retrolateral view; **C** Distal part of procursus, prolateral view; **D** Distal part of procursus, prolateral-ventral view; **E** Distal part of procursus, dorsal view. Scale bars: 0.30 mm (**A, B**); 0.10 mm (**C–E**).

The new species resembles *Pholcus nagasakiensis* Strand, 1918 (Fig. 6 and Fig. 5; Huber (2011): 440, figs. 1947, 1973, 1974, 2084, 2085) by having a similar uncus (Fig. 3C) and epigyne (Fig. 3A), but it can be distinguished by the distal apophysis of the procursus as wide as long (da in Fig. 4C vs. 2× longer than wide, da in Fig. 6C), by the membranous edge of the procursus 2× longer than wide (me in Fig. 4C vs. 4× longer than wide, me in Fig. 6C), by the male chelicerae without frontal apophyses (Fig. 3D vs. present, fa in Fig. 5D), by the vulval pore plates relatively close to each other, with straight inner margin and curved outer margin (pp in Fig. 3B vs. widely separated or with curved inner margin and straight outer margin, pp in Fig. 5B and Huber 2011: figs. 1974, 2085) and by the brown marks of the carapace covering only 1/3 of the carapace (Fig. 3E and G vs. covering the carapace nearly completely, Fig. 5E and G).

#### Etymology

The specific name refers to the cave near whose entrance the new species was collected; noun in apposition.

#### Distribution

Ryukyu Archipelago (endemic to Kume-jima Island; Fig. 1).

#### Biology

The species was found in crevices on rock walls in a shaded and humid doline at one of the entrances of Yajiya-gama Cave.

### Pholcus nagasakiensis

Strand, 1918

CBE48C50-2B09-510A-A8B0-78A4310374CE

https://wsc.nmbe.ch/spec-data/33187/taxon

#### Materials

- **Type status:** Other material. **Occurrence:** recordedBy: Francesco Ballarin; individualCount: 6; sex: 3 males, 3 females; lifeStage: 6 adults; occurrenceID: 4C7F5204-73DF-5AF1-A403-FB3BD1D5BA12; **Taxon:** order: Araneae; family: Pholcidae; genus: *Pholcus*; **Location:** island: Okinawa-jima Island; stateProvince: Okinawa Prefecture; county: Kunigami District; municipality: Kunigami Village; verbatimLocality: Yona; verbatimElevation: 46 m; verbatimLatitude: 26.76051°N; verbatimLongitude: 128.21636°E; **Event:** samplingProtocol: by hand; year: 2024; month: 9; day: 2; **Record Level:** institutionCode: SYNU-Ar00525–530

#### Description

See Huber (2011).

#### Diagnosis

The species resembles *Pholcus yajiyagama* sp. nov. (Fig. 3 and Fig. 4) by having a similar uncus (Fig. 5C) and epigyne (Fig. 5A), but it can be distinguished by the distal apophysis of procursus 2× longer than wide (da in Fig. 6C vs. as wide as long, da in Fig. 4C), by the membranous edge of procursus 4× longer than wide (me in Fig. 6C vs. 2× longer than wide, me in Fig. 4C), by the male chelicerae with frontal apophyses (Fig. 5D vs. absent, fa in Fig. 3D), by the vulval pore plates widely separated or with curved inner margin and straight outer margin (pp in Fig. 5B and Huber 2011: figs. 1974, 2085 vs. relatively close to each other, with straight inner margin and curved outer margin, pp in Fig. 3B) and by the brown marks of carapace nearly covering the entire carapace (Fig. 5E and G vs. covering only 1/3 of the carapace, Fig. 3E and G).

#### Distribution

Kyushu Island, across the whole Ryukyu Archipelago and the Diaoyu Islands of China (Fig. 1) (JSC 2025).

#### Biology

The species was collected on the underside of the overhang on rocky cliffs along a humid and shaded trail.

### Pholcus otomi

Huber, 2011

E9DA9504-7D68-5C3E-80A7-941DADED13F3

https://wsc.nmbe.ch/spec-data/33199/taxon

#### Materials

- **Type status:** Other material. **Occurrence:** recordedBy: Francesco Ballarin; individualCount: 3; sex: 1 male, 2 females; lifeStage: 3 adults; occurrenceID: 8143657B-6B13-55B0-9421-3756C0534A48; **Taxon:** order: Araneae; family: Pholcidae; genus: *Pholcus*; **Location:** island: Iriomote-jima Island; stateProvince: Okinawa Prefecture; verbatimLocality: Uehara, Kuura caves system; verbatimElevation: 39 m; verbatimLatitude: 24.40102°N; verbatimLongitude: 123.84888°E; **Event:** samplingProtocol: by hand; year: 2022; month: 9; day: 21; **Record Level:** institutionCode: SYNU-Ar00531–533

#### Description

See Huber (2011).

#### Diagnosis

**Figure 7.**
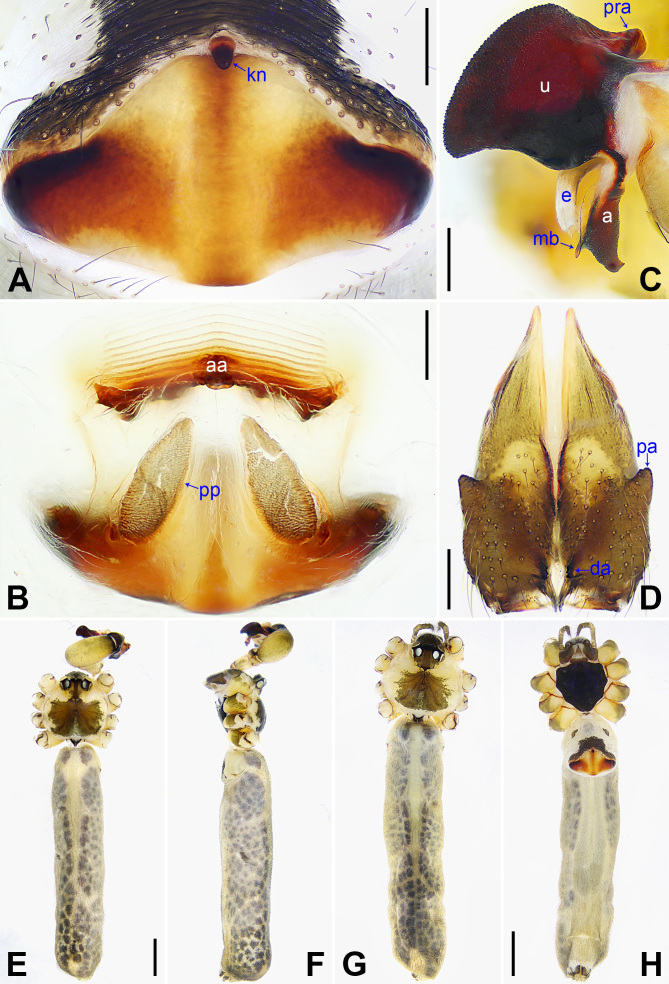
*Pholcus otomi* Huber, 2011, male (**C–F**) and female (**A, B, G, H**). **A** Epigyne, ventral view; **B** Vulva, dorsal view; **C** Bulbal apophyses, prolateral view; **D** Chelicerae, frontal view; **E, G** Habitus, dorsal view; **F** Habitus, lateral view; **H** Habitus, ventral view. Scale bars: 0.20 mm (**A–D**); 1.00 mm (**E–H**).

**Figure 8.**
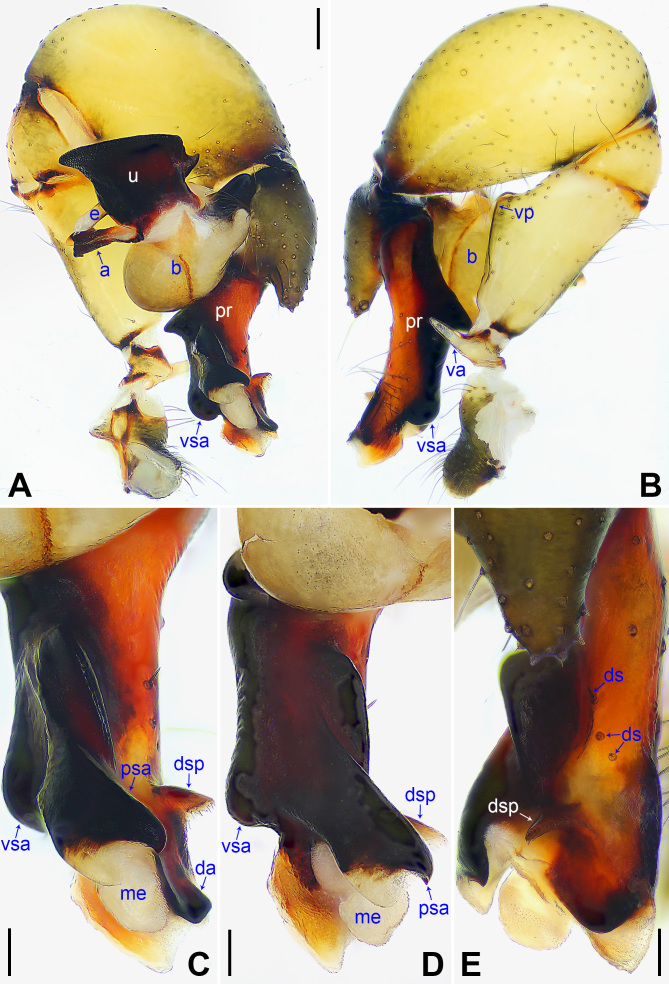
*Pholcus otomi* Huber, 2011, male. **A** Palp, prolateral view; **B** Palp, retrolateral view; **C** Distal part of procursus, prolateral view; **D** Distal part of procursus, prolateral-ventral view; **E** Distal part of procursus, dorsal view. Scale bars: 0.20 mm (**A, B**); 0.10 mm (**C–E**).

The species resembles *Pholcus yajiyagama* sp. nov. (Fig. 4 and Fig. 3) by having a similar uncus (Fig. 7C), male chelicerae (Fig. 7D) and epigyne (Fig. 7A), but it can be distinguished by the procursus with prolatero-subdistal apophysis (psa in Fig. 8C and D vs. absent, Fig. 4C) and ventro-subdistal apophysis (vsa in Fig. 8A–D vs. absent, Fig. 4A–D), by the vulval pore plates anteriorly pointed and posteriorly blunt (pp in Fig. 7B vs. nearly elliptic, Fig. 3B) and by the brown marks of the carapace nearly covering the entire carapace (Fig. 7E and G vs. covering only 1/3 of the carapace, Fig. 3E and G).

#### Distribution

Ryukyu Archipelago (Kume-jima, Iriomote-jima, Ishigaki-jima and Yonaguni-jima Islands; Fig. 1) (JSC 2025).

#### Biology

The species was found in crevices on rock walls at the entrances and twilight areas of shallow caves.

## Supplementary Material

XML Treatment for Pholcus yajiyagama

XML Treatment for Pholcus nagasakiensis

XML Treatment for Pholcus otomi

## Figures and Tables

**Table 1.** Voucher specimen information.

**Species**	**Voucher code**	**GenBank accession number**
*P. nagasakiensis*	W420	PZ280342
	W421	PZ280343
*P. otomi*	W422	PZ280344
	W423	PZ280345
*P. yajiyagama* sp. nov.	W424	PZ280346
	W425	PZ280347
	W426	PZ280348
*P. paralinzhou*	y046	MW721825
*P. taishan*	y133	MW721826

**Table 2.** Average uncorrected p-distances (below diagonal) and K2P distances (above diagonal) amongst the species discussed in this study. The maximum p-distances within each species are shown in bold on the diagonal.

	*P. nagasakiensis*	*P. otomi*	*P. yajiyagama* sp. nov.
*P. nagasakiensis*	**0.002**	0.118	0.062
*P. otomi*	0.108	**0**	0.113
*P. yajiyagama* sp. nov.	0.059	0.104	**0**
